# Serum LH level prior to progestin administration is significant on pregnancy and live birth in programmed frozen-thawed embryo transfer cycles

**DOI:** 10.3389/fendo.2023.1293576

**Published:** 2023-10-19

**Authors:** Ismail Guler, Erhan Demirdag, Munire F. C. Akdulum, Mert Polat, Ahmet Erdem, Mehmet Erdem

**Affiliations:** Gazi University School of Medicine, Department of Obstetrics and Gynecology, Ankara, Türkiye

**Keywords:** frozen-thawed embryo transfer, luteinizing hormone, estrogen, artificial endometrial preparation, programmed cycle

## Abstract

**Purpose:**

To evaluate the impact of serum LH levels prior to progestin administration on the outcomes of programmed frozen-thawed embryo transfer (FET) cycles.

**Methods:**

Retrospective cohort study was conducted to compare the treatment outcomes between four groups based on the 25 percentiles of serum LH levels before progestin administration in 596 cycles of 518 patients undergoing artificial endometrial preparation protocols for FET. Primary outcome measures were ongoing and live birth rates. Secondary outcome measures were the pregnancy rates, clinical pregnancy rates, and pregnancy loss rates.

**Results:**

The trends in clinical pregnancy (CPR) and live birth rates (LBR) increased from the first to the fourth quartile (Q1 to Q4) of serum LH levels prior to progestin administration (37,0% to 48,3%, *p* = 0.042, and 22.6% to 39.5%, respectively, *p* = 0.003). Pregnancy loss rates (PLR) were higher in group Q1, although the difference was not statistically significant. Based on a multivariate logistic regression analysis, a low serum LH level before progestin initiation was found to be the most significant predictor associated with a negative effect on live birth (OR: 0,421, 95% CI 0,178 – 0,994, *p*=0,048). The day of estrogen initiation was significantly correlated with serum LH levels and quartiles of serum LH levels before progestin administration (r=0,200, *p*=0,015 and r=0,215, *p*=0,009, respectively).

**Conclusion:**

The serum LH level prior to progestin administration significantly affects pregnancy and live birth rates in patients undergoing an artificial endometrial preparation protocol for FET. LH monitoring should be incorporated into the follow-up, in addition to assessing endometrial thickness and morphology in artificial FET cycles.

## Introduction

Due to advances in vitrification methods, the elimination of OHSS, and increased rates of preimplantation genetic testing, frozen-thawed embryo transfers (FETs) are being performed more prevalent than ever worldwide (1). So, ensuring that the endometrium is ready for the implantation of frozen-thawed embryos is crucial. Natural cycles or modified natural cycles by the trigger of ovulation, letrozole ovulation induction, and artificial endometrial preparation protocols can be used for the timing of FET. The artificial FET cycle, using estrogen alone first and then its combination with progestins to represent proliferative and secretory phases of the endometrium, is a suitable protocol, especially for oligo-anovulatory patients. It provides comparable pregnancy rates and flexibility in clinics dealing with a high volume of IVF cycles (2). FET in an artificial protocol is usually scheduled after the endometrium with a trilaminar pattern is detected to be more than 7mm thick with estrogen treatment (3). In addition, some variations of the artificial protocol to optimize treatment outcomes include down-regulation with GnRH agonists and starting doses of estrogen in a fixed or incremental fashion, with or without monitoring levels of serum steroid hormones.

In physiology, FSH selects the follicular cohort in the last few days of the previous cycle, and then stimulates follicular growth and estrogen synthesis from granulosa cells, which helps to proliferate the endometrium (4). In the follicular phase, increased estrogen levels first inhibit luteinizing hormone (LH) and then stimulate an LH surge after reaching and remaining above a critical level. Increased LH around midcycle has several well-known gonadal effects by binding to its LH/hCG receptor (LHCG-R), including ovulation of a mature oocyte, stimulation of androgen synthesis in theca cells, and production of progesterone from the corpus luteum to promote secretory changes in the endometrium for possible embryo implantation. LHCG-Rs have also been shown in many extragonadal tissues, including the endometrium in both its epithelial and stromal cells, although their exact roles have yet to be identified. However, preliminary studies suggest that LH and endometrial LHCG-R may affect implantation, pregnancy maintenance (5), endometrial vascular development, and the modulation of uterine receptivity (6).

In some artificial FET cycles without desensitization, synthetic estrogen increases serum LH levels just before progestin initiation, like what happens in a natural midcycle. Only a few clinical trials have assessed the importance of increased LH levels before progestin supplementation in artificial FET cycles, revealing that this is associated with similar (7, 8) or improved outcomes (9). This context may also explain better pregnancy outcomes in artificial FET cycles with unplanned, spontaneous follicular growth and ovulation (10).

Although the timing of progestin initiation and FET in artificial cycles after a period of estrogen treatment were mainly based on the detection of sufficient endometrial thickness and trilaminar morphology, the importance of LH levels just before the progestin initiation may change daily practice. Therefore, we aimed to investigate the effect of serum LH levels before progestin administration on the outcomes of artificial FET cycles.

## Materials and methods

We reviewed the records of 2508 THAW – ET cycles in the IVF Unit of Gazi University School of Medicine and Novaart IVF Center between January 28, 2014, and October 14, 2022. In this retrospective study, we included 596 cycles of 518 patients with an artificial endometrial preparation protocol without desensitization. Exclusion criteria included having protocols for frozen embryo transfer (FET) other than programmed cycles. Cycles involving hypogonadotropic patients, as well as those that underwent desensitization and were canceled before embryo transfer due to endometrium-related factors such as polyps, fluid, or irregularities, elevated progesterone levels, embryo degeneration after thawing, and any patient-related factors identified during the treatment follow-up period, were also excluded. Treatment outcomes were compared between four groups regarding 25 percentiles of serum LH levels before progestin administration. Institutional Review Board (IRB) approval was obtained from The Institutional Review Board and Ethics Committee of Gazi University School of Medicine.

In the study, all oocytes were fertilized with intracytoplasmic sperm injection (ICSI), and embryos were cryopreserved with the vitrification method. In the artificial endometrial preparation protocol for transferring vitrified and thawed embryos, patients started estradiol treatment (Estrofem 2mg, Novo Nordisk^®^, Istanbul, Turkey) two or three times daily, depending on the clinician’s preference, during their 2^nd^-4^th^ of menstrual cycles after the baseline evaluation. Patients were re-evaluated by transvaginal ultrasonography 6-7 days later to check the endometrium. If endometrial thickness was detected as ≥7mm with a trilaminar pattern, serum LH, estradiol, and progestin levels were measured. Then, initiation of vaginal progestin (Crinone 8% gel 90mg, qd, Merck^®^, İstanbul, Turkey, or Progestan capsule 200mg, 2q12h, Koçak Farma ^®^, İstanbul, Turkey) was planned within a day. If the thickness and morphology of the endometrium were insufficient, the dose and duration of estradiol were readjusted accordingly. Patients continued to be followed at 2-3 days intervals until the endometrium reached the ≥7mm thickness for the initiation of progestin. Although estrogen therapy was extended with the goal of achieving an endometrial thickness of ≥7mm before initiating progesterone, in cases where the endometrium did not reach 7mm, embryo transfer was also performed for patients who achieved an endometrial thickness of 6mm or more. If serum progesterone level was already elevated at the time of the decision to start progestin administration or if the endometrial thickness remained too thin < 6mm, the cycles were canceled. The duration of progestin before FET was 3 or 5 days based on the age of cleavage or blastocyst stage embryos, respectively. On the day of planned FET, after the quality of thawed embryos was re-assessed based on the Istanbul consensus (11), embryo transfer was performed with a catheter (Wallace; CooperSurgical^®^, Ballerup, Denmark) under the guidance of suprapubic pelvic ultrasonography. The maximum number of transferred embryos was 2, depending on legal regulation. A pregnancy test was measured on the 14^th^ day after FET. The rate of positivity of pregnancy test and visible intrauterine gestational sac with ultrasonography or pathology result of products of conception after an abortion indicate pregnancy and clinical pregnancy rates, respectively. An ongoing pregnancy is defined as a live fetus beyond the 20^th^ gestational week, and a live birth refers to a baby who has survived for at least one week after birth.

The data were analyzed with Statistical Package for Social Sciences (SPSS, Version 28, IBM, Chicago, IL, USA). Skewness, kurtosis, and Kolmogorov-Smirnov tests were used as normality tests. One-Way ANOVA and chi-squared tests were used to compare parametric and categorical data between groups based on 25 percentiles of serum LH levels before progestin initiation. Multivariate logistic regression analysis was performed to identify the most significant variables in predicting live birth. Primary outcome measures were live birth rate (LBR) and ongoing pregnancy rate (OPR). Secondary outcome measures included pregnancy rate (PR), clinical pregnancy rate (CPR), biochemical pregnancy loss rate, and clinical pregnancy loss rate. *p* < 0.05 was used for statistical significance.

## Results

Within the study period, among the 599 cases who received programmed frozen-thawed embryo transfer cycle, we identified 55 cycles out of 741 (7,4%) that were canceled for various reasons. Causes of cycle cancellation included endometrium-related factors (such as a remaining thickness <6mm, polyp, fluid, or irregularity) (n=22, 2,9%), elevated progesterone (n=11, 1,4%), degeneration of embryos after thawing (n=8, 1%), and various patient-related factors (n=14, 1,8%) detected during the follow-up period of treatment. Cycles in which embryo transfer was canceled for these reasons, as well as those with an absence of LH levels before progesterone initiation, were excluded from the analysis. Finally, data from 596 cycles of 518 patients were included for analysis in this study.

The baseline characteristics of cases and cycle outcomes of the FET with artificial endometrial preparation are listed in **Table 1**. The median duration of Estrogen administration before progestin initiation was 8 days.

**Table 1:** Clinical and hormonal characteristics and cycle outcomes of cases in the study.

Variable		Data
Age (years)		31,9 ± 5,1
Duration of infertility (years)		5,05 ± 3,75
BMI (kg/m^2^)		24,4 ± 4,5
Diagnosis of Infertility	Unexplained	40,5%
	Female Factor	27%
	Male Factor	26,1%
	Both Female & Male Factor	6,4%
Etiology of Female Infertility	Unexplained	67,6%
	Ovulatory Factor	24,9%
	Tubal Factor	4,8%
	Decreased Ovarian Reserve	2,7%
Duration of Estrogen administration before progestin initiation (days)^*^		8 (7, 10)
On the day of Progestin initiation,	serum LH (mIU/mL)	17,17 ± 9,41
	serum Estradiol (pg/ml)	228,45 ± 108,18
	endometrial thickness	10,08 ± 3,95
Number of Transferred Embryos		1,6 ± 0,4
Age of Transferred Embryos	Cleavage	29,5%
	Blastocyst	70,5%
Quality of Transferred Embryos	Moderate & Good	83,7%
	Poor Quality	16,3%
Outcomes per cycle (n, Rate)	Pregnancy Rate	318, 53,4%
	Clinical Pregnancy Rate	245, 41,1%
	Ongoing Pregnancy Rate	193, 32,4%
	Live Birth Rate	172, 28,9%

Data are mean ± standard deviation, ^*^ median (Q1, Q3) or rate %, BMI, body mass index; AFC, antral follicle count; FSH, follicle stimulating hormone; E2, estradiol; LH, Luteinizing hormone.

**Table 2** presents the 25 percentiles of serum LH values prior to progestin initiation, while **Table 3** compares characteristics and cycle outcomes between groups based on these values. The group with serum LH <25^th^ percentile had significantly lower rates of live birth, ongoing pregnancy, clinical pregnancy, and pregnancy, compared to other quartiles. Rates of PR, CPR, OPR, and LBR increased from Q1 to Q4 of serum LH level before progestin administration. However, biochemical, and clinical pregnancy loss rates were similar among groups. Early initiation of estrogen treatment was significantly associated with a higher likelihood of being in the first quartile of serum LH before progestin administration. There was a significant correlation between the day of estrogen initiation and both the serum LH levels and quartiles of serum LH level prior to progestin administration (r=0,200, *p*=0,015 and r=0,215, *p*=0,009, respectively).

**Table 2:** Values of 25 percentiles of serum LH prior to the progestin initiation.

	25^th^	50^th^	75^th^
Serum LH (mIU/mL)	10	15,88	22,21

**Table 3:** Comparison of demographic variables and cycle outcomes between quartiles based on 25 percentiles of serum LH prior to the progestin initiation.

			1^ST^ quartile	2^nd^ quartile	3^rd^ quartile	4^th^ quartile	*p*
Age (years)			31,3 ± 4,8	31,8 ± 5,1	33,0 ± 5,3	31,3 ± 5,0	**0,011**
BMI (kg/m^2^)			25,8 ± 4,9	24,0 ± 4,2	23,9 ± 4,5	23,6 ± 4,2	**0,000**
Infertility period (years)			5,2 ± 3,7	5,4 ± 4,4	4,9 ± 3,4	4,5 ± 3,2	0,224
Basal AFC			20,0 ± 8,0	17,5 ± 6,8	18,5 ± 6,5	18,6 ± 6,7	0,506
Cycle day of estrogen initiation		Day 2	39,1%	31,6%	31%	20%	**0,009**
		Day 3	34,8%	31,6%	31%	25,6%	
		Day 4	26,1%	36,8%	37,9%	45,7%	
Prior to starting progestin,		LH (mIU/mL)	7,09 ± 2,0	12,6 ± 1,7	18,7 ± 1,9	30,07 ± 7,3	**0,000**
		E2 (pg/ml)	233,0 ± 118,8	218,8 ± 100,9	235,9 ± 108,4	226,3 ± 104,3	0,525
		Progesterone (ng/mL) ^*^	0,26 (0,13, 0,50)	0,3 (0,13, 0,70)	0,3 (0,15, 0,76)	0,3 (0,16, 0,60)	0,393
		Endometrial Thickness	9,9 ± 1,7	10,0 ± 1,5	10,4 ± 7,3	9,8 ± 1,74	0,649
Number of transferred embryos		1 (n)	36,3% (53/146)	34,9% (53/152)	36,2% (54/149)	40,3% (60/149)	0,790
		2 (n)	63,7% (93/146)	65,1% (99/152)	63,8% (95/149)	59,7% (89/149)
Age of transferred embryos		Cleavage (n)	29,9% (43/144)	34,7% (52/150)	26,0% (38/146)	27,3% (39/143)	0,373
		Blastocyst (n)	70,1%(101/144)	65,3% (98/150)	74,0% (108/146)	72,7%(104/143)
Quality of transferred embryos		Moderate & Good (n)	84,1% (69/82)	85,7% (72/84)	81,5% (66/81)	83,3% (60/72)	0,905
		Poor (n)	15,9% (13/82)	14,3% (12/84)	18,5% (15/81)	16,7% (12/72)
Primary Outcomes							
Live Birth Rate, (n)			22,6%, (33/146)	26,3%, (40/152)	28,2%, (42/149)	39,5%, (57/149)	**0,003**
Ongoing Pregnancy Rate, (n)			24%, (35/146)	32,9%, (50/152)	30,9%, (46/149)	41,6%, (62/149)	**0,003**
Secondary Outcomes							
Clinical Pregnancy Rate, (n)			37,0%, (54/146)	38,2%, (58/152)	40,9%, (61/149)	48,3%, (72/149)	**0,042**
Pregnancy Rate, (n)			45,9%, (67/146)	49,3%, (75/152)	59,7%, (89/149)	58,4%, (87/149)	**0,009**
Biochemical Pregnancy Loss Rate, (n)			7,5%, (11/146)	9,2%, (14/152)	14,8%, (22/149)	8,7%, (13/149)	0,166
Clinical Pregnancy Loss Rate,			12,3%, (18/146)	5,9%, (9/152)	10,1%, (15/149)	7,4%, (11/149)	0,216

Data are mean ± standard deviation, ^*^ median (Q1, Q3) or rate (%). One Way ANOVA or chi-squared test was used for comparisons as appropriate. BMI, body mass index; AFC, antral follicle count; FSH, follicle stimulating hormone; LH, luteinizing hormone; E2, estradiol. Statistically significant *p* values were written bold.

According to the results of the multivariable logistic regression analysis, the most significant variable for predicting live birth was a low level of serum LH (<25th percentile) prior to progestin start (OR: 0,421, 95% CI 0,178 – 0,994, *p*=0,048), which outperformed other factors, including age, BMI, endometrial thickness prior to starting progestin, number of transferred embryos, embryo age, and quality, **Table 4**.

**Table 4:** Multivariate logistic regression analysis of possible predictors for live birth in frozen thawed embryo transfer cycles with artificial endometrial preparation.

Variable		Odds Ratio	95% Confidence Interval	*p*
Age		1,006	0,950 – 1,065	0,838
BMI		0,957	0,890 – 1,029	0,237
LH levels prior to starting progestin^*^	1^st^ quarter	0,421	0,178 – 0,994	**0,048**
	2^nd^ quarter	0,719	0,334 – 1,547	0,410
	3^rd^ quarter	0,649	0,297 – 1,419	0,184
	4^th^ quarter	reference		
Embryo Quality		1,384	0,582 – 3,294	0,462
Embryo Age		1,270	0,694 – 2,325	0,438
Endometrial thickness prior to starting progestin		1,027	0,855 – 1,232	0,779
Number of transferred Embryos		1,367	0,721 – 2,591	0,338

^*^Categorical Covariate, BMI, body mass index.

Statistically significant *p* value was written bold.

## Discussion

Observations from daily clinical practice suggest that higher levels of LH upon progestin initiation may result in increased pregnancy rates during frozen embryo transfer cycles with an artificial endometrial preparation protocol, despite a limited number of relevant reports in the literature. Therefore, this study was conducted to evaluate the impact of serum LH levels prior to progestin administration on the outcomes of FET cycles that use an artificial endometrial preparation protocol without desensitization. Our data indicates that higher serum LH levels prior to progestin initiation for FET were associated with increased rates of clinical pregnancy and live birth. Furthermore, being in the first quartile of LH levels prior to progestin initiation was found to be a significant negative predictor for live birth in FET cycles with artificial endometrial preparation protocol. Additionally, this study is unique in that it evaluates the impact of the cycle day of estrogen therapy initiation on LH levels prior to progestin administration. Our findings suggest that initiating estrogen therapy on cycle day 2 or 3, as opposed to cycle day 4, was associated with lower serum LH levels prior to progestin administration.

The role of LH levels on FET outcomes during artificial cycles has been evaluated in only a few studies. In the first study, which evaluated the role of LH levels on FET outcomes during artificial cycles, three study groups were established based on the 0-25th, 25th-75th, and 75th-100th percentiles of LH levels on day 14 of the artificial cycle. The results showed statistically similar pregnancy rates per ET among the groups, but CPRs increased from 12% to 16% from group 1 to group 3 (7). In the second study, which had a different design, the authors examined the role of LH rise, as measured by a doubling of LH levels on the 10^th^ day of estrogen administration, in artificial FET cycles. They found that LH rise occurred in approximately 2/3 of cycles, but this did not significantly impact cycle outcomes (8). The third study reported that the group in the first quartile, based on the 25^th^ percentile of serum LH levels before the initiation of progestin, had a significantly lower live birth rate (LBR) as the primary outcome after an artificial (FET) cycle (9). Additionally, pregnancy loss rates were found to be significantly higher in the first and second quartiles compared to the other quartiles. Consistent with the previous study, both pregnancy and live birth rates increased from the first through fourth quartile of serum LH levels prior to the day of progestin initiation in our study. Also, being in the first quartile predicted a negative effect on live birth when other confounders were considered in the regression analysis. However, in contrast, pregnancy loss rates did not differ significantly among the study groups.

This study and the previous trial mentioned above showed that LH levels before the day of progestin administration may have implications for implantation in women undergoing FET cycles. Although we do not know exactly how the mid-cycle increase in serum LH affects implantation, in addition to its well-known effects on ovulation and oocyte maturation, there is evidence suggesting possible mechanisms. In molecular studies, LH has been shown to modulate implantation by binding to endometrial LHCG-Rs that are strongly expressed during the implantation window in mice (12). Increased expression of human endometrial LHCG-R in the mid-luteal phase has also been demonstrated, suggesting cross-talk between hCG and LH to enhance endometrial receptivity (13). LH and LHCG-R may also regulate endometrial angiogenesis, which is important for uterine receptivity and implantation (5, 6).

A recent systematic review on endometrial preparation for FET showed that natural or modified natural cycles are superior to artificial FET protocols (14). A significantly lower pregnancy loss rate was also found in natural and letrozole FETs compared to programmed FETs (15). It can be proposed that the superiority of natural and modified natural protocols over artificial FET cycles is related to LH function. All natural, modified natural, and letrozole FETs depend on the LH rise, but not all artificial FET cycles are associated with an LH rise. In a study based on the theory of endometrial LHCG-R, subcutaneous injections of low-dose hCG during the proliferative phase in artificial FET cycles, with and without desensitization, were successful in increasing endometrial thickness and receptivity in patients with a history of recurrent implantation failure and thin endometrium (16). These clinical results also indirectly support the effectiveness of LH in implantation. In this context, it can be speculated that some methods that reduce LH, such as pre-cycle oral contraceptive use or starting the second artificial cycle without a break, may also be associated with adverse pregnancy outcomes. Low pregnancy rates in GnRH agonist-triggered fresh cycles may be related to an early and rapid rise and fall in endogenous LH levels, which could be directly detrimental to the endometrium in addition to causing early luteolysis (17).

To improve outcomes based on the significant findings of this study, we can consider avoiding desensitization protocols, which can suppress LH levels. Additionally, initiating estrogen on day 4 instead of day 2 or 3 may be helpful in achieving the desired serum LH levels prior to progestin initiation, as early onset of estrogen administration may be associated with a rapid rise and subsequent fall of serum LH levels. Patients with a history of recurrent implantation failure and low serum LH levels prior to progestin initiation with preferred endometrial thickness and morphology may benefit from natural, modified natural, or letrozole ovulation induction protocols in FET cycles. Another strategy is initiating low-dose subcutaneous hCG injections concomitantly with progestin administration to activate LHCG-R in the endometrium. While no study in the literature have evaluated this, step-up estrogen administration may also offer a potential alternative to the late initiation of estrogen therapy (on the 4^th^ day instead of the 2^nd^ or 3^rd^ day of menstruation) to achieve higher levels of LH before progestin initiation. Although a recent study compared two step-up estrogen protocols to a fixed-dose estrogen regimen without desensitization, it did not assess LH levels before progestin initiation and estrogen was started on the 2^nd^ or 3^rd^ day of menstruation (18). Nevertheless, the pregnancy rates were comparable among all three groups.

In conclusion, while this study did not evaluate serum LH levels throughout the period of estrogen supplementation, low LH levels at the start of progestin administration may be indicative of an early rise in LH, which may be in a downward trend or may have already fallen and not yet risen. Nevertheless, this study, which is the second of its kind in the literature, revealed a significant association between low serum LH levels at the onset of progestin and reduced pregnancy and live birth rates in programmed frozen embryo transfer (FET) cycles. Given that early initiation of estrogen therapy may be associated with lower LH levels prior to progestin administration, delaying estrogen initiation until the 4th day of the cycle may be a beneficial strategy for improving pregnancy and live birth outcomes in frozen-thawed embryo transfer cycles.

## Data availability statement

The raw data supporting the conclusions of this article will be made available by the authors, without undue reservation.

## Ethics statement

The studies involving humans were approved by The Institutional Review Board and Ethics Committee of Gazi University School of Medicine. The studies were conducted in accordance with the local legislation and institutional requirements. The participants provided their written informed consent to participate in this study.

## Author contributions

IG: Writing – original draft, Writing – review & editing. ED: Data curation, Writing – review & editing. MA: Data curation, Writing – review & editing. MP: Data curation, Writing – review & editing. AE: Methodology, Supervision, Writing – review & editing. ME: Data curation, Methodology, Supervision, Writing – review & editing.
